# 启东市2001年-2007年肺癌生存率分析

**DOI:** 10.3779/j.issn.1009-3419.2011.01.05

**Published:** 2011-01-20

**Authors:** 健 朱, 永辉 张, 永胜 陈, 璐璐 丁, 建国 陈

**Affiliations:** 226200 启东，启东肝癌防治研究所 Qidong Liver Cancer Institute, Qidong 226200, China

**Keywords:** 肺肿瘤, 生存率, 流行病学, Lung neoplasms, Survival, Epidemiology

## Abstract

**背景与目的:**

生存率研究是反映癌症预后的一个重要指标。本研究旨在对启东2001年-2007年全人群肺癌登记病例进行生存率分析，为预后评价及防治提供依据。

**方法:**

4, 451例登记病例的生存（死亡）情况随访截止于2009年12月31日；剔除只有死亡医学证明书（death certificate only, DCO）病例，实际纳入分析4, 382例。用SURV 3.01软件计算观察生存率（observed survival, OS）及相对生存率（relative survival, RS），并应用*Hakulinen*氏似然比检验法进行统计学检验。

**结果:**

肺癌1年、3年、5年OS分别为23.73%、11.89%和10.01%，1年、3年、5年RS分别为24.86%、13.69%和12.73%。其中男性1年、3年、5年RS分别为23.70%、12.58%和11.73%，女性1年、3年、5年RS分别为27.89%、16.53%和15.21%，女性生存率高于男性，两组之间的差异具有统计学意义（χ^2^=13.77, *P*=0.032）。15岁-34岁、35岁-44岁、45岁-54岁、55岁-64岁、65岁-74岁及75^+^岁各年龄组的5年RS分别为35.46%、17.66%、11.97%、13.49%、10.61%和15.14%。启东2001年-2007年5年RS比1972年-2000年有了很大的提高。

**结论:**

启东市全人群肺癌登记病例总体生存率有了较大的提高，但与国外发达国家相比尚有一定的差距，应当继续重视肺癌的诊治研究。

## Abstract (en)

**Background and objective:**

Lung cancer is one of the most important malignancies in China. Survival rates of lung cancer on the population-based cancer registry for the years 2001-2007 in Qidong were analysed in order to provide the basis for the prognosis assessment and the control of this cancer.

**Methods:**

Total 4, 451 registered lung cancer cases was followed up to December 31st, 2009. Death certificates only (DCO) cases were excluded, leaving 4, 382 cases for survival analysis. Cumulative observed survival rate (OS) and relative survival rate (RS) were calculated using *Hakulinen's* method performed by the SURV 3.01 software developed at the Finnish Cancer Registry.

**Results:**

The 1-, 3-, and 5-year OS rates were 23.73%, 11.89%, 10.01%, and the RS rates were 24.86%, 13.69%, 12.73%, respectively. The 1-, 3-, and 5-year RS of males vs females were 23.70% *vs* 27.89%, 12.58% *vs* 16.53%, and 11.73% *vs* 15.21%, respectively, with statisitically significant differences (χ^2^=13.77, *P*=0.032). RS of age groups of 15-34, 35-44, 45-54, 55-64, 65-74 and 75^+^ were 35.46%, 17.66%, 11.97%, 13.49%, 10.61%, 15.14%, respectively. Remarkable improvement could be seen for the 5-year RS in this setting if compared with that for the years 1972-2000.

**Conclusion:**

The lung cancer survival outcomes in Qidong have been improved gradually for the past decades. Further measures on the prevention, diagnosis and treatment of lung cancer should be taken.

30多年来，我国肺癌的发病率和死亡率呈现持续上升的趋势，已上升为城市的第一位死亡原因^[1-4]^。启东市自20世纪70年代肺癌发病率、死亡率也明显上升，居全部恶性肿瘤的第二位^[5, 6]^，所造成的危害也日益严重。肺癌生存率研究是反映其预后的一个重要指标，为了解我市肺癌近期生存现状，为行政部门制定和评估肺癌的预防控制措施提供决策依据，本研究根据启东2001年-2007年以人群为基础的恶性肿瘤登记报告资料，对启东肺癌观察生存率及相对生存率作一分析。

## 1 材料与方法

### 1.1 资料来源

启东自1972年起建立了癌症登记报告系统^[5]^，该系统覆盖了启东全境、全部自然人群及全部恶性肿瘤；启东1974年还建立了全部死亡原因登记报告制度。在启东登记报告系统各级医疗卫生机构中工作的专职人员每月向上一级报告所覆盖区域内的全部新病例，同时报告病例死亡情况，即“发病”与“死亡”报告。每年年底肿瘤病例还与全死因登记病例进行核校。这些都为肺癌等恶性肿瘤最终随访结局的记录提供了保证。启东的癌症发病、死亡情况还先后收录入国际癌症研究中心及国际癌症登记协会编撰的《五大洲癌症发病率》 ^[7, 8]^及《中国恶性肿瘤发病与死亡》出版物中。本系列肺癌病例资料即来源于该癌症登记报告系统2001年-2007年资料。

### 1.2 病例随访与资料处理

在对数据收集和初步审核后，按国际疾病分类ICD-O2分类编码。应用国际癌症研究中心（International Agency for Research on Cancer, IARC）的IARC-CHECK程序，对患者的年龄、出生日期、发病日期、肺癌的亚部位和病理学类型等进行逻辑审核。对2001年-2007年资料中“尚存活”的所有肿瘤病例，于2010年上半年进行病例随访。生存时间的计算截止至2009年12月31日。

启东2001年-2007年肺癌新发病登记病例共计4, 451例（男3, 224例，女1, 227例），性别比例为2.63:1，其中经组织学确定（HV）的为747例（16.78%）。有些病例的发病日期未知，首先登记自“死亡”报告或“死亡医学证明”，则称之为“只有死亡医学证明书”（death certifcate only, DCO）病例。本系列中剔除DCO病例69例（1.55%），实际纳入本分析的为4, 382例（占98.45%），其中男性3, 175例（98.48%），女性1, 207例（98.37%）。

### 1.3 统计方法

观察生存率（observed survival, OS）指某单位时段开始时存活的个体在该时段结束时仍存活的概率，其计算公式为*S*(*t*)=*∏*(1-*d*_*j*_/*n*_*j*_)。式中*S*(*t*)为*t*年生存率，*j*为死亡或截尾的时间，*d*_*j*_为*j*时刻的暴露人口数。如观察期内有截尾，则分母用校正人口数。以人群为基础的登记资料的相对生存率（relative survival, RS）的计算，则引入了同期同性别同年龄组人群生存概率的概念，有助于不同地区不同人群的生存率资料的比较。RS为OS与同期同性别同年龄组人群寿命中生存概率之比，即：*Sc*(*t*)=*So*(*t*)/*Se*(*t*)。式中，*Sc*(*t*)为相对生存率，*So*(*t*)为观察生存率，*Se*(*t*) =*∑n*_*x*_*S*_*ex*_(*t*)/*∑n*_*x*_，其中*n*_*x*_为在*X*岁开始随访的人数，*S*_*ex*_(*t*)为*X*岁开始*t*时刻的生存概率。预期生存概率来自本所生命登记处预期寿命表，计算方法同文献^[9]^。用*Hakulinen*氏等编制的SURV 3.01软件^[10]^计算OS及RS，并应用*Hakulinen*氏似然比检验法^[11]^进行χ^2^检验，以*P* < 0.05为有统计学差异。

## 2 结果

### 2.1 肺癌观察生存率与相对生存率

启东市2001年-2007年肺癌病例总体1年、3年、5年、8年OS分别为23.73%、11.89%、10.01%和9.77%，RS分别为24.86%、13.69%、12.73%和14.63%。1年-8年各OS及RS见表 1。

**1:** 启东市2001年-2007年肺癌观察生存率与相对生存率（%） The observed survival rate and relative survival rate (%) of lung cancer in Qidong during 2001-2007

Survival year (*n*)	OS (%）	2^*^SE_*OS*_	RS (%)	2^*^SE_*RS*_
1	23.73	1.29	24.86	1.35
2	14.22	1.06	15.61	1.16
3	11.89	0.99	13.69	1.14
4	10.78	0.97	13.04	1.17
5	10.01	0.96	12.73	1.23
6	9.95	0.97	13.33	1.29
7	9.77	0.98	13.83	1.39
8	9.77	0.98	14.63	1.47
OS: observed survival rate; RS: relative survival rate.				

### 2.2 不同性别肺癌生存率

肺癌患者的男、女性1年、3年、5年OS分别为22.55%、10.83%、9.09%和26.84%、14.69%、12.40%，男、女性1年、3年、5年RS分别为23.70%、12.58%、11.73%和27.89%、16.53%、15.21%。男性*n*年OS、RS均低于女性相应生存年数的OS、RS（表 2）。女性总体RS高于男性，差异有统计学意义（χ^2^=13.77, *P*=0.032）。

**2:** 启东市2001年-2007年肺癌男女性观察生存率及相对生存率（%） The observed survival rate and relative survival rate (%) of lung cancer by sex in Qidong during 2001-2007

Survival year (*n*)	Male			Female	
	OS	RS		OS	RS
1	22.55	23.70		26.84	27.89
2	13.17	14.55		16.98	18.37
3	10.83	12.58		14.69	16.53
4	9.80	11.98		13.37	15.69
5	9.09	11.73		12.40	15.21
6	9.09	12.40		12.17	15.60
7	8.98	12.98		11.79	15.81
8	8.98	13.78		11.79	16.57

### 2.3 不同年龄组肺癌生存率

启东市2001年-2007年肺癌男女性患者OS、RS均随年龄的增加有下降趋势，但75^+^岁RS有所回升（表 3）。男性各年龄组间生存率比较差异有统计学意义（χ^2^=13.54, *P*=0.019）；女性各年龄组间生存率比较差异具有统计学意义（χ^2^=49.93, *P* < 0.001）；男女合计各年龄组间生存率比较差异具有统计学意义（χ^2^=79.35, *P* < 0.001）。

**3:** 启东市2001年-2007年肺癌各年龄组5年观察生存率及相对生存率（%） The 5-year observed survival rate and relative survival rate (%) of lung cancer by age in Qidong during 2001-2007

Age group	Male				Female				Total		
	Cases	OS	RS		Cases	OS	RS		Cases	OS	RS
15-34	6	66.67	67.12		11	18.18	18.25		17	35.29	35.46
35-44	80	21.21	21.69		62	12.83	12.95		142	17.37	17.66
45-54	299	11.51	12.01		165	11.75	12.02		464	11.56	11.97
55-64	746	12.13	13.06		232	13.95	14.55		978	12.62	13.49
65-74	1, 217	7.54	9.46		372	12.23	14.10		1, 589	8.63	10.61
75+	827	6.26	12.46		365	11.64	20.95		1, 192	7.86	15.14
Total	3, 175	9.09	11.73		1 207	12.40	15.21		4, 382	10.01	12.73

### 2.4 与启东1972年-2000年不同时期肺癌生存率的比较

表 4列出了启东市1972年-2000年中6个不同时期男女性肺癌5年OS和RS^[12]^，可看出2001年-2007年男女性肺癌的5年OS、RS均有明显提高。

**4:** 启东市不同时期男女性肺癌5年观察生存率及相对生存率的比较（%） The comparison of the 5-year observed survival rate and relative survival rate (%) of lung cancer by period and by sex in Qidong

Period^[12]^	Male			Female	
	OS	RS		OS	RS
1972-1976	3.74	4.29		6.95	7.59
1977-1981	3.81	4.44		2.61	2.88
1982-1986	4.78	5.56		4.15	4.56
1987-1991	3.70	4.32		5.58	6.16
1992-1996	4.75	5.51		6.15	6.74
1997-2000	7.87	8.87		12.87	13.95
2001-2007	9.09	11.73		12.40	15.21

## 3 讨论

目前国内恶性肿瘤生存率资料主要来源于医院临床报道，而临床报告的生存率受医院治疗水平、治疗方法及患者病情等因素的影响较大，无法反映恶性肿瘤的总体生存状况。以人群为基础的肿瘤生存资料能够反映某地区肿瘤患者的生存概况。生存率观察资料中包括完整观察资料和截尾资料，这些都已有相应的统计学方法给予解释并在软件中得以实现而不影响人群的总体生存率的评价。本资料来源于启东市癌症登记资料，包括启东市2001年-2007年所有新发肺癌患者，故分析结果可反映整个启东市全人群中近期肺癌的生存率情况，对于评价启东市肺癌防治的整体水平具有较大意义。

虽然近年来肺癌临床治疗方面有较大进展，但肺癌的5年RS仍较低，一般低于15%^[13]^。本文结果显示，启东市2001年-2007年肺癌5年OS、RS分别为10.01%与12.73%，1年生存率也只有23.73%（OS）与24.86%（RS）。由于肺癌早期诊断困难，约1/3早期肺癌患者无任何临床症状，肺癌患者误诊率高达46.8%^[14]^，因而预后较差。

启东不同性别肺癌生存率结果显示，女性各年生存率均优于男性，提示肺癌对男性的危害大于女性。而8年RS有所回升，可能由于其对应的预期生存概率有较大下降的缘故。北京曾对1990年-2000年间1, 272例原发性肺癌生存时间及影响因素进行分析^[15]^，发现男女性生存时间的影响因子不尽相同。影响因子种类的不同及影响因子的强弱不同导致了男女性在肺癌生存率上的差异。

启东肺癌观察生存率OS随年龄增加有下降趋势，说明随年龄增长，身体功能减弱，患病后预后较差，同时也反映了早期发现对于提高肺癌生存率的重要性。不同年龄组间的RS在男性、女性、男女合计中均表现出有统计学差异。肺癌生存率在不同年龄组之间的差异有不同的文献报道。如Ramalingam等^[16]^报道青年人肺癌预后优于中老年人，2年生存率分别为25.6%和23.1%（*P*=0.005），5年生存率分别为16.1%和13.4%（*P* < 0.001）；日本Mizushima等^[17]^报道肺癌患者5年生存率在30岁以下（84.6%）组高于30岁-49岁（22.5%）、50岁-69岁（30.9%）和70岁以上（7.6%）组，均与本报道一致。但Whooley等^[18]^则认为，青年人肺癌在生物学行为方面侵袭性更强、晚期患者多及预后差，与本结论不一致。笔者认为启东地区青壮年肺癌生存率相对较高的原因，可能与青年人全身状况较好、接受更积极的手术治疗的占比高有关，而中老年人治疗则相对保守。而75^+^岁年龄组的RS反而较高，可能的原因是75^+^岁年龄组死于其它原因的风险明显增大，根据RS的生存概率之比公式，因而RS相对较高。

与1972年-2000年不同时期肺癌生存率的比较显示，2001年-2007年男女性肺癌的5年OS、RS有了明显提高。这可能与医学诊断和治疗水平逐步提高、社会经济整体状况的发展和完善有关；也可能与群众防癌抗癌意识增强、早期诊断与及时治疗的肺癌患者数量增多有关。

目前国内外近期以人群为基础的肺癌生存率报道并不多见。可以检索到的国内外不同地区全人群肺癌生存率资料进行比较（表 5）。由表 5可见，不同地区肺癌5年相对生存率差距很大。国内上海^[19]^、天津^[20]^、北京^[21]^等大城市生存率水平在11%-13%，农村福建长乐^[22]^、浙江嘉善^[23]^、山东临朐^[24]^的生存率水平均不到10%。国外美国^[25]^德国^[26]^韩国^[27]^日本^[28]^国家的生存率水平在14%-23%，而乌干达Kampala^[29]^的5年RS为0。启东2001年-2007年5年RS男性为11.73%，女性为15.21%，比较可看出启东2001年-2007年肺癌患者5年生存率水平与上海、天津、北京等大城市十多年前的生存率水平相当，也高于农村地区福建长乐、浙江嘉善、山东临朐的水平，但与日本等国尚有差距。这可能与肺癌防治工作的开展情况、国家地区间肺癌诊断和治疗水平的差异、分析时段、DCO占比的差异等有关；也说明根据现有的医疗技术水平，肺癌生存率尚有较大的提高空间。

**5:** 启东市与国内外不同地区肺癌5年相对生存率的比较（%） The comparison of the 5-year relative survival rate (%) of lung cancer among Qidong and different regions

Period	Area	Male	Female
1988-1991	Shanghai^[19]^	12.0	11.3
1981-1985	Tianjin^[20]^	13.1	12.0
1987-1988	Beijing^[21]^	11.9	11.8
1988-1993	Changle^[22]^	5.5	3.9
1987-2002	Jiashan^[23]^	7.5	9.6
1993-1999	Linqu^[24]^	9.0	9.4
1973-2003	America SEER^[25]^	14.0^*^	
2000-2002	Germany Saarand^[26]^	15.4^*^	
1998-2002	Korea^[27]^	12.4	17.4
1993-1996	Japan^[28]^	20.7	27.6
1993-1997	Uganda Kampala^[29]^	0.0^*^	
^*^Male and female combined data.			

由于本资料基于人群、属农村地区资料，因而肺癌病理学诊断依据占比相对较低，为16.78%，这可能也是本文的局限性之一，但这并不影响本文的生存率结果的分析。有资料^[28]^显示手术治疗的肺癌患者生存期较长，肺癌的早期发现和早期治疗是延长患者生存期的关键因素。但由于目前尚缺乏有较高灵敏度和特异度的早期诊断和筛检手段，因此，笔者认为，在目前肺癌发病率不断上升而生存率相对较低的情况下，我们更应该从贯彻预防为主的方针着手，加大肺癌防治研究工作的力度。
